# NGS‐based targeted sequencing guides risk‐adapted and molecularly targeted therapy decisions in multiple myeloma

**DOI:** 10.1111/bjh.70111

**Published:** 2025-08-26

**Authors:** Gaurav Agarwal, Mohammad Kazeroun, Mirian Salazar, Khrystal McBean, Ananya Kannan, Jemma Larham, Sherin Varghese, Berrin Balik, Joseph Browning, Alexis Caulier, Y. L. Tracey Chan, Hannah Giles, Matthew Jenner, Bhuvan Kishore, Guy Pratt, Neil Rabin, Rebecca Robinson, Alexis Talbot, Zoé van de Wyngaert, Ke Xu, Sally Moore, Eileen Boyle, Naser Ansari‐Pour, Karthik Ramasamy, Angela Hamblin, Anjan Thakurta, Sarah Gooding

**Affiliations:** ^1^ Division of Hematology/Oncology Boston Children's Hospital, Harvard Medical School Boston Massachusetts USA; ^2^ Oxford Translational Myeloma Centre, Nuffield Department of Orthopaedics, Rheumatology and Musculoskeletal Sciences (NDORMS) University of Oxford Oxford UK; ^3^ Department of Haematology Oxford University Hospitals NHS Foundation Trust Oxford UK; ^4^ University Hospitals Birmingham NHS Trust Birmingham UK; ^5^ CRUK Clinical Trials Unit University of Birmingham Birmingham UK; ^6^ Department of Haematology University Hospital Southampton Southampton UK; ^7^ Department of Haematology University College London Hospitals NHS Foundation Trust London UK; ^8^ Department of Haematology, Hôpital Saint Louis, APHP Université Paris Cité Paris France; ^9^ Sorbonne Université, Centre de Recherche Saint‐Antoine INSERM UMRs938, Service d'Hématologie Clinique et de Thérapie Cellulaire Hôpital Saint Antoine, AP‐HP Paris France; ^10^ Department of Haematology University Hospitals Bristol and Weston NHS Foundation Trust Bristol UK; ^11^ MRC Molecular Haematology Unit, Weatherall Institute of Molecular Medicine University of Oxford Oxford UK

**Keywords:** cancer genetics, clinical cytogenetics, diagnostic haematology, fish, genetic analysis, myeloma

To the Editor,

Despite advances in therapy, patients with high‐risk multiple myeloma continue to experience poor outcomes. A proportion of these patients are not identified by current risk stratification systems, such as the Revised International Staging System (R‐ISS), and only identified to be high risk in retrospect, on early disease progression and death.^1^ More accurate identification of high‐risk patients prospectively at diagnosis would improve prognostication, patient counselling and tailoring of front‐line therapy. To this end, a variety of molecular profiling tools have been investigated to define high‐risk status, including gene expression panels.^2^ Since 2014, the International Myeloma Working Group (IMWG) has recommended fluorescence in situ hybridization (FISH) as standard of care for detecting genetic lesions.^3^, ^4^ However, FISH detects only a limited subset of features and fails to explain poor outcomes in many patients. Recently, targeted next‐generation sequencing (NGS)‐based diagnostics offering expanded genomic insights have become increasingly accessible in clinical settings.^5^ Yet, whether clinicians value this increased scope and their potential to alter clinical decision‐making in multiple myeloma remains largely unexplored.

To address this, we sought to compare head‐to‐head utility of FISH with the Myeloma Genome Project targeted sequencing panel (MGPP). MGPP is a myeloma‐specific targeted region NGS assay designed to detect oncogenic drivers and actionable targets, covering 228 genes, 6 translocation regions and 56 copy number abnormalities (CNAs).^5^ MGPP requires fewer CD138^+^ myeloma cells than FISH (approximately 100 000 vs. over 500 000 cells) and captures more prognostic variants than the limited number of chromosome‐level features assessed by FISH [commonly 1q21 gain, 1p32 deletion, 17p13 deletion, t(4;14), t(14;16), t(11;14), t(14;20)]. Moreover, MGPP achieves a higher depth (minimum 200× for translocations and 500× for small variants) than is routinely accepted in clinical grade WGS, enabling variant detection at small subclone level,^6^ and remains easier to scale in a clinically useful time frame. MGPP identifies emerging high‐risk genomic features such as bi‐allelic 1p32 deletion and other double and triple hit features (the co‐occurrence of any ≥2 or ≥3 high‐risk features, comprising t(4;14) t(14;16), t(14;20), 17p deletion, *TP53* mutation, 1q21 gain and 1p32 deletion).^7^ However, the utility of MGPP in supporting clinical risk designation and therapy choice, when compared with FISH in newly diagnosed myeloma, is unknown.

In a prospective cohort, we performed both MGPP and FISH in routine clinical workflows, in matched samples from 50 newly diagnosed myeloma patients. The median age was 70 years, 54% were transplant eligible and the cohort included patients with R‐ISS 1 (26%), R‐ISS 2 (42%) and R‐ISS 3 (30%) disease (Table S1). ISO‐accredited FISH analyses were performed in NHS laboratories for all patients, as per standard of care. In addition, MGPP was performed using 100 ng DNA extracted from bone marrow (BM)‐derived CD138^+^ cells and matched germline peripheral blood samples, as previously described.^5^ Somatic mutations, CNAs and translocations were called with a custom pipeline using specified QC metrics (Supplemental Methods).

We first compared the quantity and resolution of genomic data reported by MGPP versus FISH. MGPP had greater technical success, reporting successfully in 100% of samples with sufficient BM material, compared with 82% success of FISH across matched BM samples (*n* = 9 assay failures) (Figure 1A). Moreover, MGPP reported more genomic features than FISH, detecting 364 vs. 54 total somatic variants across 128 vs. 11 regions respectively. While MGPP detected nearly all variants reported by FISH, equivalent loci were additionally captured at greater resolution (Figure 1B). Notably, MGPP was able to detect 17p13 deletion (*N* = 1) and 1p32 deletions (*N* = 8) that were missed by FISH in matched samples, likely due to lower coverage of myeloma cells (typically 100–200) by FISH. In one sample, FISH detected t(11;14) which was not reported by MGPP, consistent with the expected levels of discrepancy between two orthogonal methodologies, as previously reported.^5^ Furthermore, MGPP identified additional prognostic genomic aberrations that were beyond the scope of FISH, including *MYC* translocations (*N* = 3) and *TP53* mutations (*N* = 3). These findings are consistent with MGPP enabling improved genomic resolution compared with FISH,^5^ at both equivalent and an expanded set of prognostic loci.

**FIGURE 1:**
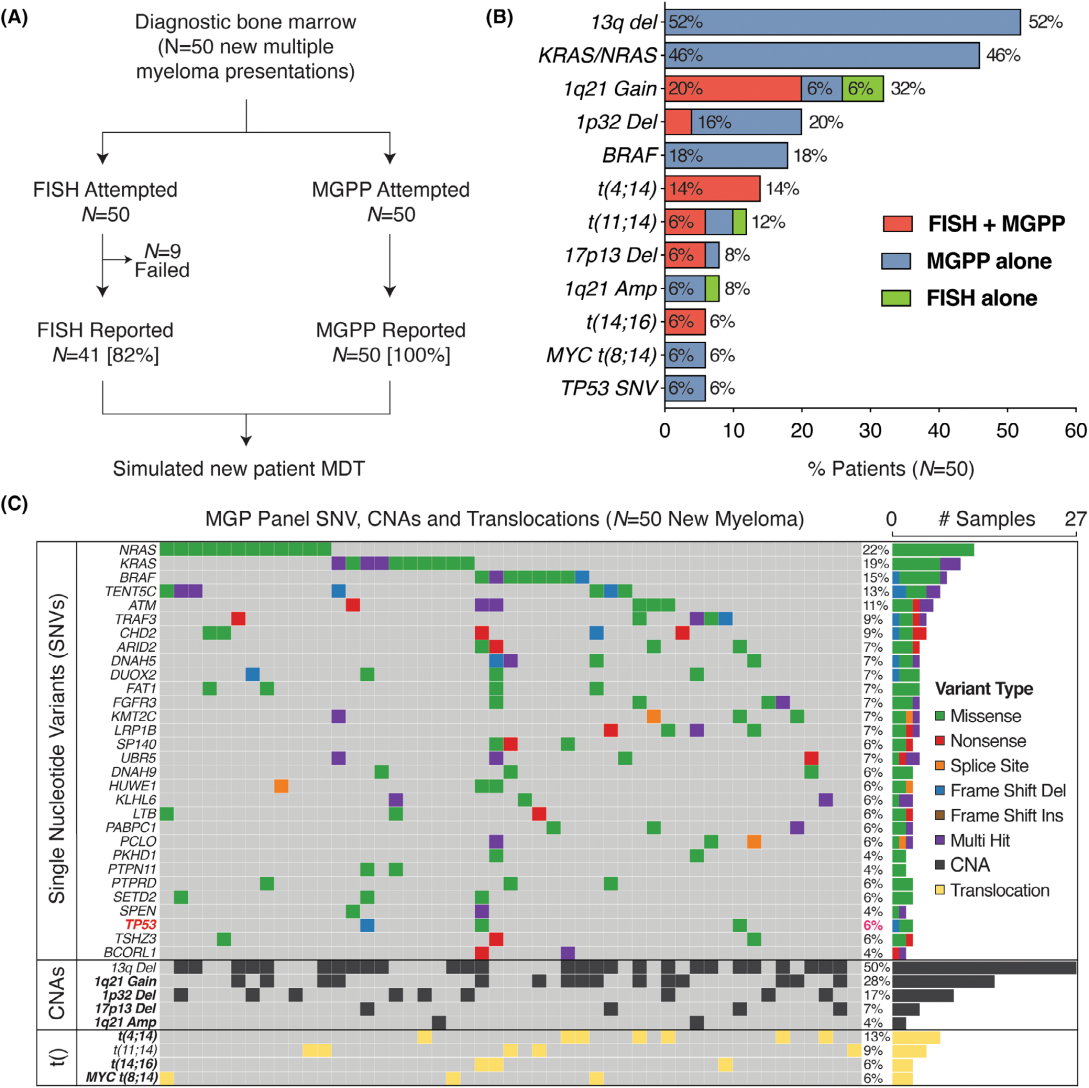
MGPP identifies prognostic genomic markers undetected by FISH in newly diagnosed myeloma. (A) FISH and MGPP were attempted and reported from matched bone marrow samples of 50 newly diagnosed myeloma patients, prior to a simulated new patient multidisciplinary team (MDT) meeting. (B) Copy number abnormalities (CNAs), translocations and a selection of single nucleotide variants (SNVs) detected by FISH, MGPP or both assays. (C) Oncoplot showing individual level set of CNAs, translocations and selection of top SNVs detected by MGPP. FISH, fluorescence in situ hybridization; MGPP, Myeloma Genome Project Panel.

To assess the clinical utility of MGPP, we conducted a multicentre simulated multidisciplinary team (MDT) meeting. Fifteen blinded myeloma specialist clinicians (from three European countries) independently reviewed each patient's case presented twice (with clinical data known at diagnosis, and either FISH or MGPP data), in a randomized order (Figure 2A). Panellists completed a structured pro forma, in which they were asked, based on the available information, to designate low‐ versus high‐risk status,^1^ standard versus intensified induction regimen,^2^ (for transplant‐eligible patients only) single versus tandem transplant^3^ and consider a set of specific genetic lesion‐targeted therapies at relapse if available (from a list of those already in phase 2 or above clinical trials)^4^ (Figure 2B). The majority consensus was taken as the MDT outcome for each question, which were then compared between FISH and MGPP for each case.

**FIGURE 2:**
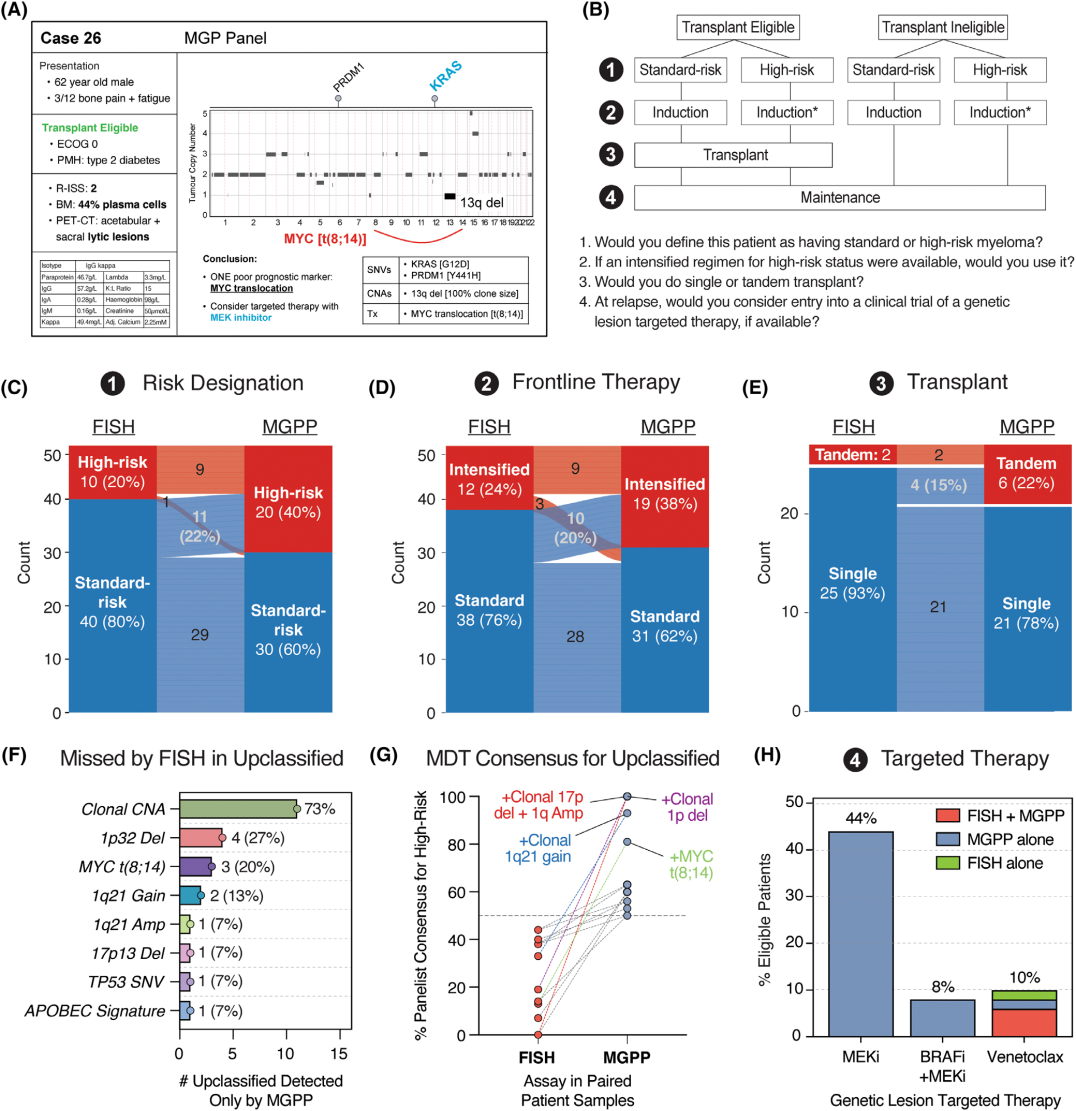
MGPP alters clinician‐led risk designation and therapy choice in newly diagnosed myeloma patients. (A) Representative case presented to multidisciplinary team (MDT) panellists of 15 specialist myeloma clinicians, containing clinical information and either FISH or MGPP report. (B) Panellists were asked up to four questions per case, relating to risk designation and treatment. (C) Change in clinician‐led risk designation (standard risk or high risk) with either FISH or MGPP. (D) Change in front‐line therapy choice (standard vs. intensified regimen) with FISH or MGPP. (E) Change in decision for single versus tandem transplant, with FISH or MGPP, among *N* = 27 transplant‐eligible patients. (F) Genomic features identified by MGPP but not FISH, among 15 patients upclassified for risk status and/or therapy choice(s). (G) Percent of MDT panellists designating high‐risk status for patients upclassified from a consensus standard risk (FISH) to high risk (MGPP). (H) Proportion of patients identified as eligible for a molecularly targeted therapy. FISH, fluorescence in situ hybridization; MGPP, Myeloma Genome Project Panel.

In a head‐to‐head comparison with FISH, MGPP altered risk designation and treatment decisions at diagnosis. The clinician‐led MDT reclassified 11 (22%) patients designated as standard risk by FISH to high‐risk status when presented with MGPP (Figure 2C). Moreover, clinicians opted for intensification of induction therapy in 24% (FISH) vs. 38% (MGPP) patients (Figure 2D) and tandem autologous transplant in 7% (FISH) vs. 22% (MGPP) of transplant‐eligible patients (Figure 2E). As clinical data were otherwise identical, these findings suggest MGPP directly influences treatment decisions, enabling an identification of high‐risk patients missed by FISH and facilitating risk‐adapted therapy.

We next investigated whether up‐classification was associated with specific genetic lesions. Of 15 patients upclassified by myeloma clinicians for risk status and/or treatment, 10/15 (66%) had at least one poor prognostic marker identified by MGPP that was missed by FISH (Figure 2F). In 5/15 (33%) patients, MGPP identified the same CNA as FISH but also confirmed it as a clonal event. Importantly, upclassified patients carried *MYC* translocations (*N* = 3) and several CNAs—including 1p32 deletion (*N* = 4), 1q21 gain/amp (*N* = 3) and 17p13 deletion (*N* = 1)—none of which were detected by FISH. Of three patients with previously unknown *TP53* mutations detected by MGPP, one was upclassified and the other two carried additional high‐risk markers identified by FISH and did not have their designation changed. Cases with the most striking consensus shift were enriched for poor prognostic markers (Figure 2G), suggesting that the identification of these previously missed genetic lesions was associated with altered treatment decisions by MDT panel members.

Finally, we asked whether the extra genetic resolution of the MGPP may identify opportunities for targeted therapy (Figure 2H). MDT panel members were asked whether, at relapse, they would consider entry into a clinical trial of a genetic lesion‐targeted therapy, if eligible. We considered three such drugs that are currently in at least phase 2 trial for patients with relapsed myeloma—venetoclax [for t(11;14)],^8^ MEK inhibitor [for *KRAS*/*NRAS*‐mutated myeloma]^9^ or combined BRAF and MEK inhibitors [for *BRAF*‐mutated myeloma].^10^ Of these, FISH was able to identify 8% of patients as eligible for venetoclax. In addition, MGPP further identified eligibility for MEK inhibitors (44%) and BRAF+MEK inhibitors (8%), comprising a striking proportion of patients. In all cases, the MDT consensus was to consider these agents, if available on a trial in the relapse setting. These data suggest that, in addition to the identification of prognostic markers missed by FISH, MGPP can also identify therapeutic opportunities for personalized targeted therapy, enhancing the identification of patients that may benefit from trial entry.

As it becomes more feasible to provide increased genetic information on cancers in a timely and clinically deployable manner, it is critical to ask whether this may be practice changing for clinicians in routine care. Clinical decision‐making will drive reimbursement for these panels and funding for diagnostic infrastructure to accommodate the increased complexity. Indeed, studies are refining the predictive power of genomic correlates of high‐risk multiple myeloma at diagnosis,^11^ and increasingly asking whether treatment intensification according to upfront high‐risk designation improves outcomes.^3^, ^12^ To the best of our knowledge, this is the first report to ask the utility of targeted NGS panel‐delivered genetics in clinical decision‐making for real‐world myeloma patients. Importantly, we demonstrate that MGPP can be implemented into routine NHS workflows, with a 14‐day clinical turnaround time. We demonstrate an improved assay success rate, increased sensitivity at equivalent loci and an extended array of prognostic features that are missed by current FISH standard of care. Revised IMWG criteria for high‐risk designation in myeloma^13^ include *TP53* mutation as a criterion for high‐risk disease and—for the first time—suggest the use of NGS for routine molecular profiling; we anticipate the increased implementation of such panels into clinical infrastructure for myeloma diagnostics will be required to accommodate this testing. Finally, genomics‐based analysis of relapsed patients with MGPP may enable a deeper understanding of clonal evolution in individual patients to guide therapy sequencing.

Our results now warrant the implementation of MGPP in larger cohorts. While we have shown that MGPP alters clinical risk designation, this pilot study did not have the power to determine if this associates with long‐term outcomes (Figure S1). There remains a lack of consensus on appropriate intensification regimens, or how their use should be determined, with mixed evidence supporting the value of risk‐adapted treatment.^3^, ^14^, ^15^ To systematically investigate this, we are now deploying MGPP into the ongoing UK‐MRA RADAR trial (trial registration number ISRCTN46841867),^14^ to assess whether shifting myeloma genomic risk stratification to NGS platforms across NHS infrastructure is beneficial to patient care and outcomes. The opportunities from this would be twofold. First, given that MGPP captures all recurrently mutated regions of the myeloma genome, its increased rollout will provide an opportunity for long‐term observational studies to expand our knowledge of prognostic markers in myeloma. In turn, large‐scale implementation of MGPP in the United Kingdom has the potential to alter long‐term outcomes for people with multiple myeloma by appropriately designating their risk features and tailoring treatment prospectively at diagnosis.

## AUTHOR CONTRIBUTIONS

G.A., K.R., A.Th. and S.G. conceptualized the study. G.A., A.K., J.L. and E.B. collated clinical data. G.A., M.S., K.M., J.L. and S.V. collected and processed patient samples. M.K. and N.A.‐P. performed somatic variant calling. B.B., J.B., A.C., Y.L.T.C., H.G., M.J., B.K., G.P., N.R., R.R., A.Ta., Z.W., K.X., S.M. and E.B. were panellists for the simulated multidisciplinary team meeting. G.A. analysed and presented the data. A.H. provided oversight of NHS laboratory testing. G.A. and S.G. wrote the paper, with input from all authors.

## FUNDING INFORMATION

This work was supported by the NIHR Oxford Biomedical Research Centre (BRC) grant (G.A., K.R. and S.G.) and the Oxford Translational Myeloma Centre (A.T.). B.B. is funded by the Turkish Society of Haematology with the International Support Programme for Haematology Residents and Consultants. S.G. is funded by a Cancer Research UK Fellowship grant RCCCSF‐Nov21\100004 and works in an UKRI MRC‐funded unit.

## CONFLICT OF INTEREST STATEMENT

The authors have no conflicts of interest to disclose.

## ETHICS STATEMENT

Bone marrow samples were obtained from UK Research Ethics Committee‐approved tissue biobanks Oxford Radcliffe Biobank (South Central—Oxford C REC: 19/SC/0173) and HaemBio (REC Reference: 17/SC/0572. Sponsor: University of Oxford).

## PATIENT CONSENT STATEMENT

Informed consent was obtained from all subjects involved in the study. Patient data are anonymized.

## Supporting information


Data S1.

## Data Availability

Sequencing datasets in process of being deposited publicly. Please contact lead author for update/access.
