# Supplementary material for: NGS‐based targeted sequencing guides risk‐adapted and molecularly targeted therapy decisions in multiple myeloma
Source: Br J Haematol. 2025 Aug 26;207(5):2167–72. doi: 10.1111/bjh.70111 (PMC12624159; doi:10.1111/bjh.70111)
Supplement: Supplementary file 1 — Data S1. [file BJH-207-2167-s001.docx]

**Supplementary Table 1. Baseline patient demographics and disease characteristics.**

| **Patient Demographics & Disease Characteristics** | | **Value** |
| --- | --- | --- |
| *N* | | 50 |
| Age, years (median [IQR]) | | 70 [58, 75] |
| Sex (%) | Male | 37 (74%) |
|  | Female | 13 (26%) |
| ECOG | 0 | 26 (52%) |
|  | 1 | 19 (38%) |
|  | 2 | 3 (6%) |
|  | 3 | 2 (4%) |
| Transplant Eligible | Yes | 27 (54%) |
|  | No | 23 (46%) |
| R-ISS (%) | R-ISS 1 | 13 (26%) |
|  | R-ISS 2 | 21 (42%) |
|  | R-ISS 3 | 15 (30%) |
|  | Unknown | 1 (2%) |
| Myeloma Isotype | IgG | 32 (64%) |
|  | IgA | 10 (20%) |
|  | Light chain | 4 (8%) |
|  | Unknown | 4 (8%) |
| Tumour burden (median [IQR]) | % BM plasma cells | 39.0 [23.5, 56.5] |
| Lytic lesions | Yes | 34 (68%) |
|  | No | 16 (32%) |
| Serum biomarkers (median [IQR]) | Haemoglobin, g/L | 115.5 [95.5, 131.0] |
|  | Creatinine, g/L | 73.5 [65.0, 107.8] |
|  | Adjusted calcium, g/L | 2.45 [2.31, 2.50] |
|  | Beta-2 microglobulin, mg/L | 3.10 [2.35, 6.60] |
|  | Albumin, g/L | 3.4 [2.9, 3.9] |
|  | LDH, U/L | 202 [150, 222] |

**Supp. Figure 1. Progression-free survival by FISH or MGPP-designated risk status.**

Progression-free survival (PFS) by clinician-led risk stratification from time of diagnosis, based on consensus multidisciplinary team (MDT) classification on standard or high-risk status, given clinical cases with either: (**A**) FISH (A); (**B**) MGPP.

**(C)** PFS of patients stratified as consensus standard-risk (green), consensus high-risk (red), or standard-risk with FISH and upclassified to high-risk with MGPP (purple).

FISH = fluorescence in-situ hybridization; MGPP = Myeloma Genome Project Panel. Log-rank test.


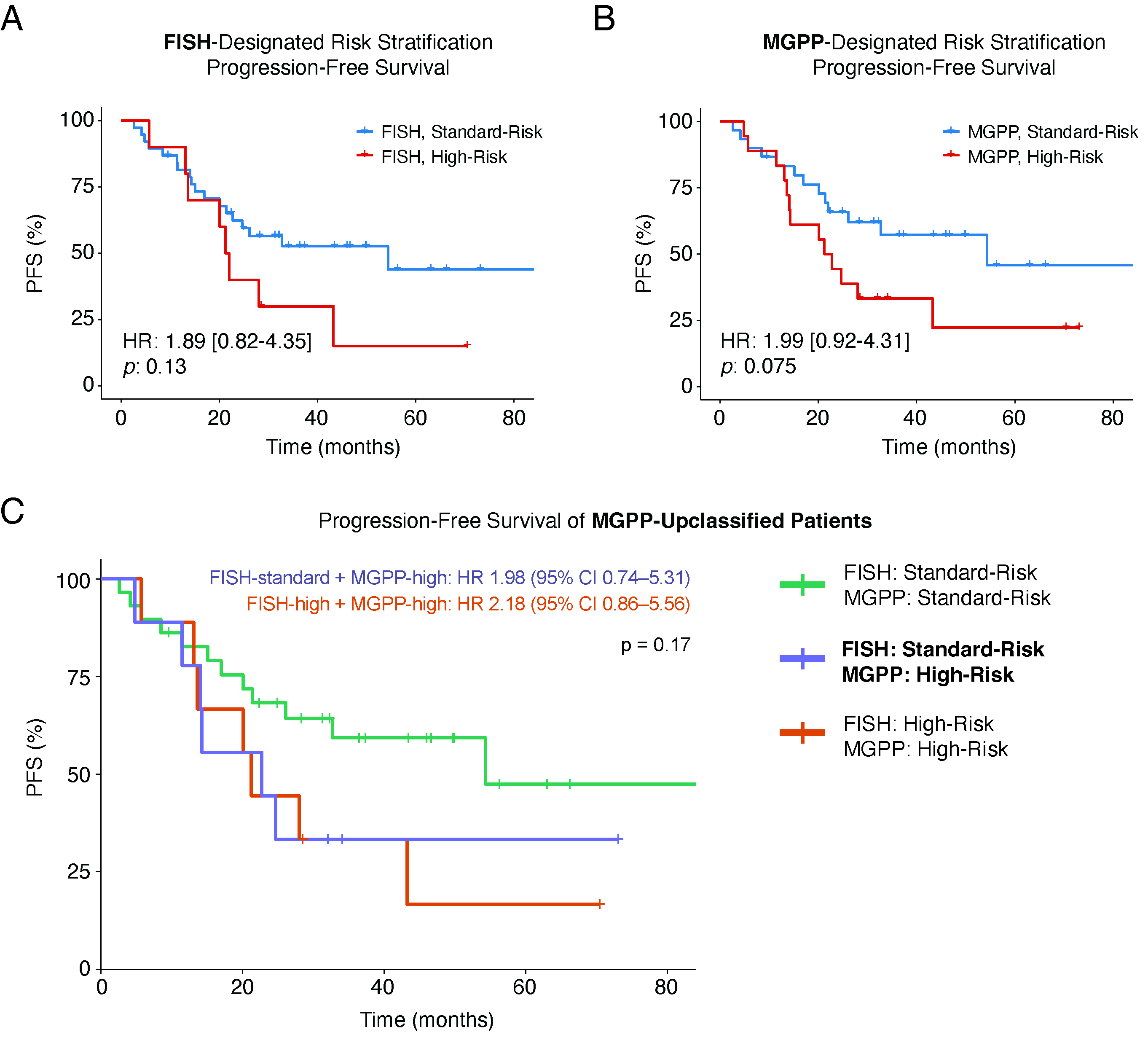


**Supplemental Methods**

Bone marrow samples were obtained from UK Research Ethics Committee-approved tissue biobanks Oxford Radcliffe Biobank (South Central - Oxford C REC: 19/SC/0173) and HaemBio (REC Reference: 17/SC/0572. Sponsor: University of Oxford). CD138^+^ multiple myeloma cells were magnetic bead enriched (EasySep #17887, StemCell Technologies) from diagnostic bone marrow aspirates of *N*=50 newly diagnosed myeloma patients. Matched peripheral blood was collected for germline calling. DNA from bone marrow and peripheral blood was extracted using standard column purification methods.

Targeted sequencing with the Myeloma Genome Project Panel (MGPP) was performed using 100 ng each of tumour and germline DNA, on 290 genes and chromosomal regions that had previously been shown to be relevant to the biology, prognosis, and treatment of multiple myeloma (2). The panel was divided into a translocation panel and a mutation/copy number panel to provide high depth coverage for mutation analysis while providing lower depth sequencing of translocation regions. Libraries were prepared with custom probe sets for mutation and translocation panels (v2.1; IRN 1000008523 and 1000008533, respectively; KAPA HyperCap reagents, Roche), as described: <https://sequencing.roche.com/content/dam/diagnostics_microsites/sequencing/master-blueprint/en/resources/pdfs/brochures/kapa-hypercap-workflow-mc--04078.pdf>)

Sequencing was conducted on an Illumina NovaSeq with paired-end 150 base-pair reads under NHS Genomic Laboratory Hub provision. In line with clinical standards, the minimum sequencing depth requirement (after removal of PCR repeats) was 200x for translocations (usually clonal) and 500x for small variants (often sub-clonal).

A standardised bioinformatics analysis pipeline was consistently applied to all collected samples. Burrows-Wheeler Aligner (BWA) mem (v. 0.7.17) was used for alignment to the human reference genome (GRCh38/hg38). Marking duplicate reads and base recalibration was performed in line with the GATK best practice workflow with Picard MarkDuplicates and ApplyBQSR respectively. Subsequently, short variants including single-nucleotide variants (SNVs) and indels were called using GATK Mutect2 (gatk:4.4.0.0). Variant annotation was carried out using ANNOVAR (v. 20191024) and 10% variant allele frequency (VAF) threshold was applied to filter artefacts. Intra- and inter-chromosomal rearrangements were detected using Manta (v. 1.6.0) with default settings and the exome flag specified. To detect CNA events, we developed a purity-informed segmentation approach by analysing normalised depth between paired tumour and control/germline samples. Briefly, coverage of each base pair sequenced in the panel was obtained using alleleCounter with default mapping and base quality parameters (MAPQ>35, BASEQ>20). LogR was calculated as the ratio of the coverage in the tumour to that in the germline for each nucleotide position and then normalised against their genome-wide mean value. Regions of chromosomal deletion and gain were then identified using segmentation of LogR by using piecewise constant fitting (PCF). The LogR of each segment was then used to estimate the total copy number (TCN) per segment by calculating expected LogR values for clonal TCNs 1 to 10 and taking account of the purity of the tumour while allowing for subclonal states (defined as significant deviation from expected clonal states). A minimum of 30% cancer cell fraction (CCF) was used as a cut-off to distinguish noise in LogR variance from genuine subclonal CNA events.

**Data availability statement**

Sequencing datasets in process of being deposited publicly. Please contact lead author for update / access.

**Author contributions**

G.A., K.R., A.Th. and S.G. conceptualized the study. G.A., A.K., J.L. and E.B. collated clinical data. G.A., M.S., K.M., J.L. and S.V. collected and processed patient samples. M.K. and N.A-P. performed somatic variant calling. B.B., J.B., A.C., Y.L.T.C., H.G., M.J., B.K., G.P., N.R., R.R., A.Ta., Z.W., K.X., S.M. and E.B. were panelists for the simulated multidisciplinary team meeting. G.A. analyzed and presented the data. A.H. provided oversight of NHS laboratory testing. G.A. and S.G. wrote the paper, with input from all authors.

**Acknowledgements**

We are grateful to the patients who donated clinical samples, and the NHS South Central Genomic Laboratory Hub for performing sample sequencing.

**Funding information**

This work was supported by an Oxford NIHR Biomedical Research Centre (BRC) grant (G.A., K.R., S.G.) and the Oxford Translational Myeloma Centre (A.T.). B.B. is funded by the Turkish Society of Haematology with the International Support Programme for Haematology Residents and Consultants. S.G. is funded by a CRUK Fellowship grant RCCCSF-Nov21\100004 and works in an UKRI MRC-funded unit.

**Conflicts of Interest**

The authors have no conflicts of interest to disclose.

**Ethics statement**

Bone marrow samples were obtained from UK Research Ethics Committee-approved tissue biobanks Oxford Radcliffe Biobank (South Central - Oxford C REC: 19/SC/0173) and HaemBio (REC Reference: 17/SC/0572. Sponsor: University of Oxford).

**Patient consent statement**

Informed consent was obtained from all subjects involved in the study. Patient data are anonymized.
